# Harmonisation of variables names prior to conducting statistical analyses with multiple datasets: an automated approach

**DOI:** 10.1186/1472-6947-11-33

**Published:** 2011-05-19

**Authors:** Xavier Bosch-Capblanch

**Affiliations:** 1 Swiss Tropical and Public Health Institute, Socinstrasse 57, Basel 4051, Switzerland; 2 University of Basel, Basel, Switzerland

## Abstract

**Background:**

Data requirements by governments, donors and the international community to measure health and development achievements have increased in the last decade. Datasets produced in surveys conducted in several countries and years are often combined to analyse time trends and geographical patterns of demographic and health related indicators. However, since not all datasets have the same structure, variables definitions and codes, they have to be harmonised prior to submitting them to the statistical analyses. Manually searching, renaming and recoding variables are extremely tedious and prone to errors tasks, overall when the number of datasets and variables are large. This article presents an automated approach to harmonise variables names across several datasets, which optimises the search of variables, minimises manual inputs and reduces the risk of error.

**Results:**

Three consecutive algorithms are applied iteratively to search for each variable of interest for the analyses in all datasets. The first search (A) captures particular cases that could not be solved in an automated way in the search iterations; the second search (B) is run if search A produced no hits and identifies variables the labels of which contain certain key terms defined by the user. If this search produces no hits, a third one (C) is run to retrieve variables which have been identified in other surveys, as an illustration. For each variable of interest, the outputs of these engines can be (O1) a single best matching variable is found, (O2) more than one matching variable is found or (O3) not matching variables are found. Output O2 is solved by user judgement. Examples using four variables are presented showing that the searches have a 100% sensitivity and specificity after a second iteration.

**Conclusion:**

Efficient and tested automated algorithms should be used to support the harmonisation process needed to analyse multiple datasets. This is especially relevant when the numbers of datasets or variables to be included are large.

## Background

Data requirements to measure health related indicators have increased in the last decade. On the one hand, governments and the international community need to monitor progress towards health and development targets[1] in order to inform decisions at national and international levels (e.g. the Millennium Development Goals); whereas on the other, donor governments, international agencies, Non-Governmental Organizations and Global Health Initiatives, which are accountable to their constituencies, are expected to attribute measurable changes to the support they provide[2]. Geographical comparisons and time trends can show patterns or ranges of changes (or their absence) in health related indicators. These types of analyses encompass a considerable breadth of data from different geographical areas and timeframes.

Nation-wide, representative, household surveys, such as Demographic and Health Surveys (DHS)[3] and Multi-Indicator Cluster Surveys (MICS)[4], have been carried out in around one hundred countries since the 1980's and 90's respectively. Basic analyses usually are undertaken by the organisations or institutions implementing the surveys and disseminated in the form of reports, tables or interactive queries[5,6]. However, in order to undertake other specific analyses at sub-national, national and global levels, individual subject datasets (or 'microdata') have to be used[7,8]. This is possible as most of the DHS and MICS datasets are freely available after registration[9]. The availability of DHS and MICS datasets provides an excellent opportunity for conducting statistical analyses with outputs across multiple surveys.

Designs, data collection and data management procedures are relatively standard for the same type of survey (DHS and MICS); however, there are differences in dataset structures between DHS and MICS and within each of these types of surveys (e.g. diverse variables names for the same variable and/or different coding of the same variables). Therefore, prior to conducting analyses involving several datasets, it is paramount that data structures are fully harmonised (i.e. renaming variables and recoding their values, where appropriate).

Harmonisation can take place at several stages in surveys development and analysis: at design stage (harmonising measurements -inputs- or statistical outputs) or after data has been collected ('ex-post')[10]. Standards[11] and regulations[12] are available to conduct surveys. However, too often surveys may not follow standards because standards do not necessarily apply to all types of surveys, or because surveys were carried out before standards were defined, or countries had their own preferences, or because surveys have a local scope. The focus of harmonisation along this article is 'ex-post' harmonisation, after data has been collected.

When the numbers of datasets and variables to be harmonised are small, dataset structures can be handled manually. When the number of datasets and variables are large (e.g. there were more than 250 DHS and MICS datasets, with several hundreds of variables in some of our analyses), harmonisation of dataset structures becomes tedious, as well as time-consuming and extremely prone to error.

This article presents an automated approach to harmonise dataset structures, which optimises the search of variables, minimises manual inputs and reduces the risk of error. This approach is to be implemented prior to any statistical analysis involving multiple datasets and allows running the analyses without programme halts due to errors of inconsistency. Examples will be drawn from recent analyses of vaccination variables based on DHS and MICS. The approach described can be applied to any group of datasets and for any type of analysis. The statistical software used was Stata IC 10 (StataCorp LP, USA) running on Windows XP Professional, version 2002, SP3. Table 1 describes the technical terms used along the article.

**Table 1:** Terminology of variable categorisation.

Terms	Description	Examples
Dataset	Stata data files have the extension '.dta' containing the data to be analysed.	ke_DHS_41.dta

Observation	Each of the subjects for which data in the form of variables have been collected.	Observations are numerated from 1 to the total number of observations

Variable	Each item of information for each subject in a dataset.	Vaccination against the third dose of DTP

Variable name	The name given to the variable, which is used for data management and analyses.	DTP3, hi8, im

Variable label	A free text to explain the information contained in variable.	'DTP3 vaccination status of the child'

Variables of interest	Variables defined by the user, which are to be included in the analyses, and which have to be searched for in the datasets	'DTP3', if the vaccination status of the third DTP dose will be used in the analyses

Candidate variables	Existing variables in the datasets (e.g. surveys) which need to be renamed to the names of the variables of interest to become harmonised	'im8', 'im15'... (these are variables pointing at the vaccination status of the third DTP dose)

Value	The numerical, logical, date, time or string information for a given variable in an observation.	1, 2, 9

Value label	Text label attached to each possible value of variable (in a categorical variable or certain values of non-categorical variables).	1: not vaccinated 2: vaccinated 9: unknown

[Commands] (*)	Terms and expressions used in Stata to undertake data management or analytical actions.	[display], [regress], [lookfor], [codebook]

Do, do file	Files in text format that store commands and that Stata can execute in sequence.	Start.do

'Current'	The term current (applied to variables or datasets) indicates the variables being considered in a programme at run time or the datasets loaded in memory.	No example

(*) Stata commands are written in brackets all throughout this article.

### The problem

Variables names, labels, values and values labels are the four items which unambiguously identify each variable in a dataset. These four items are often inconsistent across datasets for the same variable. How can inconsistencies be solved to create fully harmonised datasets in terms of variables names, labels, values and values labels, in an automated way?

Taking the third dose of Diphtheria-Tetanus-Pertussis vaccination (DTP3), Table 2 illustrates real examples of naming variations in three surveys.

**Table 2:** Inconsistencies for the variable 'third dose of DTP vaccine' in three datasets.

Survey	DR Congo 2001	Kenya 2003	Moldova 2000
**Type**	MICS 2(*)	DHS	MICS 2(*)

**Dataset name**	dc-MICS_2-ch.dta	ke-DHS_41-ch.dta	mo-MICS_2-ch.dta

**DTP3 variable names**	dtcoq3	h7	dpt3

**DTP3 variables labels**	'enfant vaccine au dtcoq3'	'received dpt 3'	'dose 3, DTP'

**DTP3 values and** **values labels**	0 (not labelled) 1 'Oui'	0 no 1 vacc. date on card 2 reported by mother 3 vacc. marked on card 8 dk	1 vaccination card 2 mother's report 3 not vaccinated

'dk': doesn't know; 'vacc.': vaccination.

(*) There are three versions of MICS, depending on the year in which surveys have been conducted. 'dtcoq3', 'h7' and 'dpt3' are the names given to the variable DTP3 in those surveys.

The last three rows show the information that has to be harmonised across datasets prior to conducing statistical analyses using those variables.

## Implementation

In order to conduct statistical analyses across all surveys, 'dtcoq3', 'h7' and 'dpt3' (candidate variables referring to variable of interest DTP3) should have the same name and codes (Table 2). Datasets are harmonised when all variables pointing at the same content have the same name and the same range of values (and the same codes, for categorical variables); for example, when all variables carrying the information about the vaccination status of the third dose of DTP have the same name in all datasets (e.g. DTP3).

This is accomplished by identifying variables in the datasets and renaming them to harmonise names:

(a) searching, in each dataset, for candidate variables and matching those of interest (e.g. variable 'dtcoq3' in 'dc-MICS_2-ch.dta', 'h7' in 'ke-DHS_41-ch.dta' and 'dpt3' in 'mo-MICS_2-ch.dta', are all variables for the third dose of DTP).

(b) renaming matching variables with the names of the variables of interest (and recoding categorical variables, where appropriate) (e.g. renaming 'dtcoq3' to 'DTP3' in 'dc-MICS_2-ch.dta', and the same with 'h7' in 'ke-DHS_41-ch.dta' and 'dpt3' in 'mo-MICS_2-ch.dta').

These processes are supported by a master table created in Stata named '_Structure.dta' (Table 3). Columns contain all variables of interest as defined by the user. The column ('items') qualifies the information contained in each row. There are three groups of rows: (i) the first row containing the key terms for the variables of interest (see Search B below), (ii) the following rows with the values and value labels for each variables of interest as defined by the user; and (iii) the rest of the rows containing the best matching candidate variables as named in the datasets (the names of which are in the column 'Items'). In the example of Table 3, the user defined variables of interest were 'DOBcmc', 'DTP3' and 'Urb'.

**Table 3:** Partial view of Stata table ('_Structure.dta') containing examples of variables of interest.

Obser-vation	Items	DOBcmc	DTP3	Urb	(other variables of interest...) (*)
1	Key terms	Date Birth	DTP 3	Area Residence	

2	0	NA	Not vaccinated		

3	1	NA	Vaccinated recall	Rural	

4	2	NA	Vaccinated mark	Urban	

5	3	NA	Vaccinated date		

6	4	NA			

7	9	Inconsistent	Inconsistent	Inconsistent	

...	...	...	...	...	

11(+)	dc-MICS_2-ch.dta	cdob	dtcoq3	hi6	

12(+)	ke-DHS_41-ch.dta	v011	h7	v025	

13(+)	mo-MICS_2-ch.dta	NA	dpt3	hi6	

	(rest of the datasets)				

Rows headings (observation numbers) and columns headings are used by Stata to define the configuration of this table. From rows 1 to 7: values labels of the variables of interest defined by the user. From row 11 onwards, datasets files names and variables as named in those datasets corresponding to each variable of interest.

'DOBcmc', 'DTP3' and 'Urb' are the harmonised names for the variables date of birth DTP3 and area of residence respectively. The terms in each column, in rows 11 to 13, refer to the names of those variables in the datasets.

The file may contain additional fields as needed (e.g. additional information of the datasets included in the analyses) and fields can be renamed to be accommodated to other programming practices.

NA: not available (a numerical variable usually does not have codes).

(*) There are as many additional columns as variables of interest. (+) There are as many additional rows as datasets included in the project or analyses.

Based on the correspondences contained in '_Structure.dta' between variables of interest and matching variables in each dataset, variables would be renamed to names of the variables of interest. Renaming all variables (and recoding all values, where appropriate) would allow harmonise datasets.

A Stata programme captures the file names of all datasets in the data directory and creates the table '_Structure.dta' storing the file names in successive rows. The variables of interest and their key terms, which depend on the specific project or analyses to be carried out, are manually entered by the user.

### 1. Overview of the algorithm

**Figure 1:**
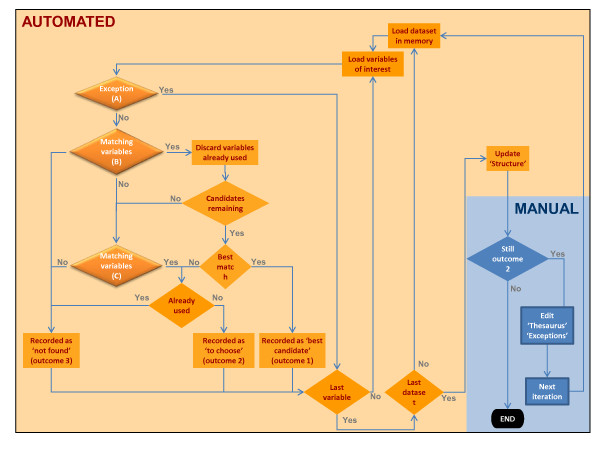
**Harmonisation processes**. In orange colours: automated processes; in blue colours: manual processes. Diamond shapes represent processes with decision points and rectangles represent actions.

Stata programmes has been written in 'do' files which (1) sequentially load datasets in memory; and for each dataset (2) sequentially retrieve the information for each variable of interest from '_Structure.dta' ('1Sub0_Datasetsvars.do' in additional file 1). See Figure 1.

The automatic loading of datasets obviates the need to write or repeatedly edit dataset names in Stata programmes when datasets are renamed, included, excluded or moved. The Stata programme with the loop engine is stored in 'Start.do' (in additional file 1).

Once a dataset is loaded, all variable labels are normalised in order to remove non-alphanumeric characters and certain words (e.g. articles, conjunctions), and to split numbers from letters (e.g. 'DTP-3 dose given to the infant' would be converted to 'DTP 3 dose given infant'; see 'fNormTxt.ado' in additional file 1). This is done to avoid mismatches when comparing strings (see below search algorithm B).

Each variable of interest will be searched in all datasets using three search strategies, described below. The outcomes of the searches can be that either there is a best matching variable (O1), that several candidates were found (O2) or that no matching variables were found (O3). Once all variables of interest have been searched in all datasets, the programme will automatically process those cases where no variable was found or where a best match existed. The cases with multiple candidate variables are solved by judgment. Then a second iteration can be launched if there are cases of unresolved variables (Figure 1).

### 2. Search engine

For each variable of interest (in each dataset) a search engine will identify candidate variables that match the variable of interest. The search engine has three sequential algorithms (A, B and C). See Figure 1, shapes with orange background. See '1_Search_Variables.do' in additional file 1.

#### Search A: exceptions

In the few instances where the search algorithms cannot automatically find a unique or best suitable matching candidate variable, ad hoc judgments will have to be made. For example, in the dataset eg-DHS_41-ch.dta (survey of Egypt 2000) there were three groups of variables pointing at the date when DTP3 was received (three variables for the day, three for the month and three for the year), all sharing the same key terms. Some variables contained the dates when DTP3 was administered and others the source of vaccination information (in vaccination variables the sources are typically: caregivers recall, mark in health card or date in health card). Search algorithms were unable to suggest a best matching candidate variable, since all 'looked' equally valid. A special judgment was needed for this exceptional case to decide either (a) which variables to use or (b) not to use any of them at all, based on a manual scrutiny of the survey dataset structure.

These types of cases are manually recorded as exceptions and have to be identified at the very start of the search algorithms because exceptions overrule automatic findings and, therefore, make superfluous any further search. All exceptions are cases that are manually 'forced' in those cases where other searches (B and C) cannot produce unambiguous results. Exceptions in terms of 'not found' can also be recorded if for any reason the user decides that none of the candidate variables is appropriate.

Exceptions are manually recorded in a dataset ('_Exceptions.dta') at the end of each iteration (see Figure 1). An exception is defined by (a) the dataset name, (b) the variable of interest, (c) the variable in the dataset that has to be renamed (or a 'not found' statement if the decision is not to use any of the candidate(s)) and (d) the rational to justify the exception.

At the beginning of the search engine, an exception will be searched using the current dataset and variable of interest in '_Exceptions.dta'. If an exception is found, there is no need to run searches B and C (the variable recorded in '_Exceptions.dta' is the one to be renamed). If there are no exceptions for the current variable of interest and dataset, search B will take place.

In Stata ('1_Search_Variables.dta'):

(a) Load in memory the dataset containing exceptions with [use '_Exceptions.dta', clear].

(b) Search for an exception with [markin] for the current variable of interest and the current dataset.

(c) If found, store variable to rename found in '_Exceptions.dta' in local macro 'SearchA' and proceed with the next variable of interest (or the next dataset if that was the last variable for the current dataset).

(d) If no exception was found, proceed to search algorithm B.

#### Search B: key terms

This is the main search algorithm and is based on the key terms defined by the user in '_Structure.dta' (see Table 3). Key terms (in the examples of Table 3: 'Date Birth', 'DTP 3' and 'Area Residence') are searched in datasets' variables names and labels. The steps in search B are implemented sequentially searching for each variable of interest, dataset by dataset (Figure 1).

The rules are:

• A variable in a dataset is considered as a matching candidate if all key terms are found in its name or label. The number of key terms has to be enough to unambiguously define the meaning of the variable but not more in order to avoid missing variables due to superfluous mismatches (a variable with many key terms will be harder to search for in the datasets; see below for details).

• A variable is considered as a best match if all key terms are found in its name or label and the variable name or label has no other terms.

• A variable that is already a candidate for another variable of interest in the current dataset cannot be considered a valid candidate variable.

There can be variations in the variable names and labels due to legitimate variations in nomenclature (e.g. DTP can be written as DPT), different languages (e.g. the French abbreviation for DTP is DTC) or ad hoc abbreviations (e.g. DTCOQ instead of DTC). To avoid that candidate variables with these variations are missed in the search, a thesaurus is used ('_Thesaurus.dta', see additional file 1) which stores variations of key terms. Entries to the thesaurus are manually performed when variations are identified in the search processes after each iteration (see Figure 1). Key terms and their variants are also normalised (see above) to ensure that there are no superfluous mismatches in the searches, such as non-alphanumeric characters, articles or conjunctions.

Table 4 shows two examples of key terms that could be associated to the variable of interest 'DTP3d' (day when the third dose of DTP3 was given) and three datasets with different variables names and labels for 'DTP3d' candidates. In the first dataset, both examples of key terms would identify variable 'im4cd' (note that the variable label contains terms in French, which will be identified through the thesaurus). In the second dataset, only the first key term will be found, because the term 'vaccination' is absent from the variable label 'dpt3 day' (note that 'vaccination' is a redundant term). Finally, in the third dataset, nothing will be found, because not all key terms are found in any of the two examples (actually, 'im' is badly labelled in the real dataset!). The key terms 'DTP, 3, day' are the best option, because they are the smallest number of terms that unambiguously identify the variable of interest. The case of Kyrgyzstan will be treated as an exception.

**Table 4:** Examples of key terms for DTP3 and search results using algorithm B.

Survey	DR Congo 2001	Kenya 2003	Kyrgyzstan 2005
	
Dataset	dc-MICS_2-ch.dta	ke-DHS_41-ch.dta	kyrgyzstan_2005-06-MICS_3-ch.dta
	
Variable name	im4cd	h7d	im*
	
Variable label	'jour vaccination dtcoq3'	'dpt 3 day'	'day'
**Key terms**			

DTP - 3 - day	Found	Found	Not found

DTP - 3 - vaccination - day	Found	Not found	Not found

Note: Kyrgyzstan 2005 provides a better example than Moldova 2000 (used in Table 4) to illustrate the results of searching key terms.

Based on the rules outlined above and using the normalised key terms and their alternatives, all variables in each dataset are scrutinised to check if names or labels contain all key terms (or their alternatives) of the current variable of interest. The variables identified become the candidate variables.

An additional check is done to ensure that none of the candidate variables has already been used for another variable of interest in the same dataset. This can happen, for example, when more than one candidate variable has common terms in their labels. For example, when the key terms for DTP3 ('DTP' and '3') are searched, the following variables in 'dc-MICS_2-ch.dta' will be retrieved: 'dtcoq3' (label: 'enfant vaccine au dtcoq3'), 'im4cd' (label: 'jour vaccination dtcoq3), 'im4cm' (label: 'mois vaccination dtcoq3') and 'im4cy' (label: 'annee vaccination dtcoq3'), because all of them contain the two key terms 'DTP' and '3' (or its variants). At runtime, it will be checked whether each of these variables are already registered in '_Structure.dta' for the current dataset. This checking will show that 'im4cd' had been assigned to the variable of interest 'DTP3d' (key terms 'DTP', '3' and 'day'), and therefore, would be dropped. Similarly for im4cm' and 'im4cy', leaving only 'dtcoq3'.

If nothing is found in search B, search C takes place, otherwise, search C is skipped.

In Stata ('1_Search_Variables.dta'):

(a) Retrieve the current candidate variable and its key terms.

(b) Normalise key terms using sequences of [subsinstr] (function created and stored as an 'ado' Stata file).

(c) Search each key term using [markin] in '_Thesaurus.dta' and retrieve all alternative names for each key term.

(d) Load into memory the dataset where to scrutinise the variables of interest, with [use].

(e) Perform an unspecific search of all terms and their variants in the dataset using [lookfor]. Keep only the variables in 'r(varlist)'. This step is not essential, but reduces execution time by keeping only those variables that have at least one single term or variant in their label.

(f) Generate the list of candidate variables with [ds] and store names in 'r(varlist)'.

(g) For each key term, search each variant in the labels of variables in 'r(varlist)'; and if one variant is found, consider such term as found. If none of the variants of a key term is found, discard the variable with [drop].

(h) Repeat the process using [ds] and producing 'r(varlist)', for each key term. When all key terms have been processed, the dataset will only have those variables that have not failed in any of the key terms searches and discards (i.e. variables that have not been dropped because contained all key terms in their labels). Note that the dataset is not saved in any of these processes and, therefore, there is no risk of permanently loosing information by dropping variables.

(i) The remaining candidate variables in the dataset are stored in the local macro 'SearchB'.

(j) Checking that candidate variables have not already been used: each matching variable in 'SearchB' is searched with [markin] in '_Structure.dta' in order to find out whether it has already been used for another variable of interest in the same dataset. In that case, the used variable(s) is(are) discarded from the local macro 'SearchB'.

(k) If local macro 'SearchB' is empty, then proceed to Search C.

#### Search C: existing variables in other datasets

Search algorithm C is taken into account if there were no matching candidate variables after searches A and B. Algorithm C is run to suggest variables that matched the current candidate variable but in other datasets (in the current dataset there were no matching variables). This is meant to be illustrative and to inform judgments, since variables found in other surveys can point at variables in the current survey which were not found due to poor labelling (e.g. in the dataset for the survey Kyrgyzstan 2005 no variable labels referring to vaccination dates contain any reference to the vaccine used). When a variable used in another dataset is identified, its existence in the current dataset is checked (otherwise, it makes no sense to capture it as a candidate); and it is discarded if not found.

This is done by looking in the column of the variable of interest in '_Structure.dta' (e.g. DTP3) to screen variables that matched the variable of interest in other surveys. In the example of Table 3: 'dtocq3', 'h7' and 'dpt3'; these are variables that matched the key terms for 'DTP3' in the datasets 'dc-MICS_2-ch.dta', 'ke-DHS_41-ch.dta' and 'mo-MICS_2-ch.dta', respectively. If, for example, 'dtocq3', which has been used in 'dc-MICS_2-ch.dta' for DTP3, does not exist in the current dataset, it would be dropped from the candidates list. The remaining variables will be considered as potential candidates in search C.

In Stata ('1_Search_Variables.dta'):

(a) '_Structure.dta' is loaded with [use].

(b) Variables corresponding to the variable of interest are stored as row names of a local matrix, with [mkmat rownames()]; this is an atypical application of matrices, which are used here to have the possibility to store a potentially large number of strings.

(c) The current dataset is loaded in memory with [use].

(d) For each candidate variable (in row names of the matrix), check whether it exists in the dataset, with [ds].

(e) If a variable is found in the current dataset it is store into the local macro 'SearchC'.

### 3. Handling the outcomes of the search algorithms A, B and C

The different possible outcomes after each variable of interest in each dataset has been processed are: (O1) one variable is found either as an exception (search A), or as a 'best' matching variable (search B); or (O2) there are several candidate variables but none of them seems to be a 'best' candidate (either from search B, or from search C if nothing was identified in B); and (O3) no single candidate variable is found in searches A and B.

For outcomes (O1) and (O3) (unique or a best variable found or nothing found), no judgements are needed: that candidate variable is to be renamed or nothing will be renamed, respectively. When a unique or best variable is identified, it could be renamed straightaway into the dataset at runtime; however, instead, it is recorded in '_Structure.dta' for keeping track and documenting the changes operated in the datasets ('_Structure.dta' can be used to describe the data used in statistical analyses).

For the other outcome (O2), a user judgement is needed to decide whether any of the variables retrieved would be the appropriate candidate to be renamed (e.g. because it is consistent with other datasets and its values are in the range of what would be expected) or all candidate variables will be dropped. In the first instance, it can be considered whether (i) the 'best' variable will be picked up in the next iteration (i.e. it had common terms with other variables and these variables have been assigned in the current iteration and therefore will not be retrieved as candidates in the next iteration), or (ii) a new entry into '_Thesaurus.dta' could capture the variable (e.g. terms in other languages), or (iii) it is considered an exception and a new entry will be recorded in '_Exceptions.dta'.

In Stata ('1_Search_Variables.dta'):

Outcomes are handled using four log files.

(a) 'LogSearch.txt' contains the detail of all searches, for all datasets and variables. It's the reference to document the processes.

(b) 'LogSearch_NoAction.txt' records datasets and variables for which there is no need to change anything; i.e. '_Structure.dta' is already updated and the searches do not suggest any change in candidate variables.

(c) 'LogSearch_CHOICE01.txt' records changes that need to be made into '_Structure.dta' as a result of the searches and which correspond to outcomes O1 and O3. '_Structure.dta' is updated using the programme 'Replace.do' which is automatically created and run after each iteration.

(d) 'LogSearch_CHOICEN.txt' records outcomes O2, where choices will have to be made (either editing '_Thesaurus.dta' or '_Exceptions.dta')

### 4. Renaming variables and recoding values in datasets

Once searches have been run for all variables of interest in all datasets, and all judgements have been made and updated into '_Structure.dta', this table will contain all information needed to rename the variables in the datasets and serves as well to document the renaming of variables. A programme in Stata ('2_Rename.do') retrieves information on variables of interest and on matching variables and executes the actual renaming statements in each dataset.

Up to this stage, values of variables have not yet been checked for consistency. Codes are used to qualify the values of categorical variables (a categorical variables is a variable that can take a limited number of values, and usually the values of such a variable have neither magnitude nor order; e.g. sex or vaccination status). Table 2 shows some examples of discrepancies in the codes available (e.g. in Moldova 2000 there were no values '0' or '8'), in the meaning of codes (e.g. '3' meant 'vaccination on card' in the survey Kenya 2003 and 'not vaccinated' in Moldova 2000) and on the labels of codes (e.g. 'reported by mother' in Kenya 2003 and 'mother's report' in Moldova 2000). On the other hand, continuous variables must have consistent units of measure.

There are several non-exclusive options to check and achieve consistent values in the variables:

1) creating listings of the same variables across datasets with the value labels of categorical variables (e.g. using [codebook], [labelbook] commands) and minimum and maximum values of continuous variables. These lists can be manually inspected or submitted to basic analyses to identify discrepancies;

2) conducting basic statistical analyses, variable by variable (e.g. checking frequency distributions);

3) devising a searching strategy similar to those used to identify variables labels (i.e. key terms, matching criteria), which could be applicable to categorical variables.

### 5. Additional considerations: iterations and error checks

The procedures described above are implemented iteratively two or three times: searches are run, outcomes O1 and O3 are automatically processed; judgments are made for outcomes O2; '_Exceptions.dta' and '_Structure.dta' are updated; variables are renamed and recoded; and then the whole process is run again. '_Structure.dta' and '_Exceptions.dta' were created only once and are used across several projects or analyses.

The first iteration will identify a certain number of variables, but will not necessarily identify 'best' variables in those cases where different variables have the same number of key terms and the same number of matching terms. This will need user judgements based on the outputs of the algorithm. Once solved, in the next iteration, the same search will identify a best variable, because the other one will have been already handled in previous iterations.

Furthermore, before the end of the first iteration, search C (i.e. matching variables in other surveys) cannot lead to any result because '_Structure.dta' will not have been updated yet; so, it is useful to run the processes at least a second time to get the suggested variables from search C, in the cases this is needed.

Iterations are also needed to adjust key terms of the variables of interest, to update the thesaurus and to optimise the string normalisation statements.

A third iteration can be run to make sure that searches give the best results (less variables are available to be renamed and therefore it is more likely to find best matches in search B and more specific outputs in search C); also to verify that the adjustments of key terms and thesaurus still give consistent outcomes.

After these iterations, is it still possible to have missed a variable? Yes. This would be the case when after harmonising variables names and labels and updating the thesaurus, search B would not produce any best hit. Then, still 'search C' would suggest variables used for the same variable of interest in other datasets. If not, then it is not possible to be completely sure whether the variable does not exist or it has been missed. The alternative would then be to search for it manually. However, the manual search would not be logically different from searches 'B' and 'C', except that manual searches would be less systematic and thorough.

## Results

Search engines were run for four variables to illustrate the typical functioning and outputs of the algorithm: vaccination status for the third dose of DTP ('DTP3'), day of administration of the third dose of DTP ('DTP3d'), date of interview ('DOI') and number of household members (HouMem). The two DTP3 variables were chosen because they offer problematic situations in which key terms for both variables overlap: 'DTP' and '3' for 'DTP3' and 'DTP', '3' and 'day' for 'DTP3d'. The other two were chosen because they contained different types of data: dates (for 'DOI') and integers (for HouMem). The programme was run with an empty '_Structure.dta', containing only the names, key terms and codes for the variables of interest and the rows for each dataset. A '_Thesaurus.dta' already containing variant terms was used. Statements to normalise labels and terms had already been written.

Table 5 shows the number of surveys with and without hits for each variable and all iterations, with the sensitivity and specificity of the searches.

**Table 5:** Results of the search algorithms showing the sensitivity and specificity of iterative searches.

Variables (key terms) Iterations		Variable exists	Variable does not exist	Total	Sensitivity (SS) Specificity (SP)	
**DTP3**		**205**	**36**	**241**		

Iteration 1	Found	196	0	**196**	**SS**	**95.6%**
		
	Not found	9	36	**45**	**SP**	**100.0%**

Iteration 2	Found	205	0	**205**	**SS**	**100.0%**
		
	Not found	0	36	**36**	**SP**	**100.0%**

**DTP3d**		**237**	**4**	**241**		

Iteration 1	Found	194	0	**194**	**SS**	**81.9%**
		
	Not found	43	4	**47**	**SP**	**100.0%**

Iteration 2	Found	237	0	**237**	**SS**	**100.0%**
		
	Not found	0	4	**4**	**SP**	**100.0%**

**DOI**		**238**	**3**	**241**		

Iteration 1	Found	234	0	**234**	**SS**	**98.3%**
		
	Not found	1	3	**4**	**SP**	**100.0%**

Iteration 2	Found	238	0	**238**	**SS**	**100.0%**
		
	Not found	0	3	**3**	**SP**	**100.0%**

**HouMem**		**221**	**20**	**241**		

Iteration 1	Found	147	4	**151**	**SS**	**66.5%**
		
	Not found	74	16	**90**	**SP**	**80.0%**

Iteration 2	Found	221	0	**221**	**SS**	**100.0%**
		
	Not found	0	20	**20**	**SP**	**100.0%**

DTP3: third dose of DTP; DTP3d: day of the third dose of DTP3; DOI: date of interview; HouMem: number of household members.

255 DHS and MICS were stored in the data directory. Of those, 14 were excluded because they were sub-national, or it was known beforehand that they did not include vaccination data. 241 were finally included. Two iterations were run for each variable, lasting between 14 to 19 minutes for each variable (for 50 variables, for example, the time needed would rise to 12 and 16 hours, respectively).

In the first iteration, 196, 194, 234 and 147 datasets had 'DTP3', 'DTP3d', 'DOI' and 'HouMem' variables uniquely identified, respectively. In the first iteration, in four datasets, wrong 'HouMem' were hit, which was due to other variables sharing the same key terms as those for 'household members'. The first iteration produced a list of datasets and variables for which there was no best match. This list was used to make judgments, dataset by dataset, about which variables could be the correct ones to match the variables of interest. In most of the cases, this was due to duplicate variables, either having the same data, or having variants of key terms (e.g. 'DTP3' alone versus 'DTP3 plus hepatitis B' vaccination; 'number of household members' versus 'identification of household members'). Decisions were taken and stored in '_Exceptions.dta', before running the second iteration. In another case (MICS3 for Belize 2006), terms in the variable label were separated with the character '\', which was not included in the normalisation statements; but this was fixed before the second iteration.

In the second iteration all searches with algorithm B produced the same number of unique or best matching variables plus the outputs from 'search A' based on the exceptions entered after the first iteration.

The thesaurus contained terms for date (2 terms), day (2), doses (3), DTP (5), household (5), interview (3), members (6) and vaccine (3).

In the first iterations, sensitivities (true positive rates) ranged from 66.5% up to 95.6%, depending on the variable. Specificities (true negative rates) were 100% in the first iteration, in all cases except for 'HouMem' due to four wrong assignments. In the second iterations, both sensitivities and specificities reached 100% (see Table 5).

Some interesting cases were found. For example, MICS3 in Cuba 2006 was the only one of the 35 MICS3 which had a DTP3 variable (this variable was missing in the remainder of MICS3). This was the outcome of the search engine and it was manually verified to confirm the finding. In another case, Kyrgyzstan 2005-6, all variables containing dates of vaccination were mislabelled (the vaccine denomination was missing in all labels).

## Discussion

An automated algorithm written in Stata programming language to harmonise the structures of multiple datasets has been presented. This algorithm addresses the need to harmonise datasets when datasets of interest have inconsistencies in the names and values of variables.

The programme has been written in Stata which was the software the author was most familiar with, and used in the statistical analyses where the algorithm was first conceptualised. The programme utilises procedures and commands which are available (or can be programmed) in other statistical and databases management software; e.g. renaming, recoding. The added value of this algorithm is that it systematically searches for matching variables and automates the decision rules to match variables in the existing datasets with the variables of interest for the analyses.

To our knowledge, there is no such algorithm built in currently used statistical or database software. In the context of the International Social Survey Programme (ISSP) a data wizard was developed to merge ISSP country data sets into one harmonized and integrated internal analysis file, by detecting and cleaning deviations versus a pre-defined standard template[13].The wizard, in a user friendly interface, addressed the technicalities of merging datasets from different sources. However, variable names would have been manually harmonised either before submitting the datasets to the wizard or in the wizard itself. The project was recently discontinued in favour of a wider approach now under development (personal communication Markus Quandt, GESIS, February 2010).

The algorithm can efficiently process one variable in three to five seconds. Running the whole programme for 50 variables and 250 datasets may take between 12 and 24 hours, depending on the performance of the computer (in a recent vaccination data analysis, this was the range of time needed to be run in 241 datasets, 1 125 574 included children and 126 variables). The programme was robust in terms of handling extremely large amounts of data and in producing the outputs. This is a substantial amount of time, but all procedures, from the loading of datasets, to the storage of outputs, is automatic, and does not require manual input during runtime. Some manual work is needed to build up the thesaurus and to generate the exceptions (see Figure 1) after each iteration. However, these tasks have to be done regardless whether searches are done manually or using an automated process like the one presented here. This will largely depend on the characteristics of the data to be harmonised. The search algorithms ensure that the manual part is supported by systematic searches. Furthermore, the information in the thesaurus and in the exceptions is needed, in any case, to document the changes made in the datasets.

Sensitivity and specificity are optimal and increase in successive iterations. This greatly depends on user skills in defining key terms and on the work out of the thesaurus and exceptions. The case of the MICS3 in Cuba 2006 illustrates the likelihood of making errors when variables are manually scrutinised. If datasets would have been manually scrutinised, it would have been very easy to assume that none of the MICS3 had the DTP3 variables and this survey would most likely have been excluded from further analyses. However, the automated approach identified this exception. In order to have full control over the processes and their outputs, manual checks are done after each iteration, which is facilitated by the log files with the outcomes of the searches.

## Conclusions

Efficient and tested automated algorithms should be used to support the harmonisation process needed to analyse multiple datasets. This is especially relevant when the numbers of datasets or variables to be included are large.

## Availability and requirements

Project name: Datasets harmonisation; Project home page: no home page; Operating system(s): platform independent; Programming language: Stata; Other requirements: Stata software, version 10, updated. Additional 'ado' files; License: none, besides Stata licence; Any restrictions to use by non-academics: none'

## Competing interests

The author declares that they have no competing interests.

## Authors' contributions

The author has conceived, designed, pilot tested and developed the programs presented here.

## Pre-publication history

The pre-publication history for this paper can be accessed here:

http://www.biomedcentral.com/1472-6947/11/33/prepub

## Supplementary Material

Additional file 1 **contains the directory and sub-directories structure needed to run the code**. This structure should not be changed to ensure the proper running of the programmes. The files in the sub-directories are: • two Stata sample datasets: Dataset_sample1.dta and Dataset_sample2.dta; • Ado Stata files: fFind.ado, fNormTxt.ado and fToMatrix.ado. • Do Stata files: 1_Search_Variables.do (the main programme), 1_Sub0_Datasetsvars.do, 1_Sub0_Thesaurus, 2_Rename.do and _Start.do (the do file which sets up Stata parameters and triggers the programme). • Stata dataset containing the names of datasets and variables: _Structure.dta. • Stata dataset containing alternative names in the searches: _Thesaurus.dta. • Stata dataset containing the exceptions used in search A: _Exceptions.dta. Click here for file

## References

[B1] Bhutta ZA Chopra M Axelson H Berman P Boerma T Bryce J Bustreo F Cavagnero E Cometto G Daelmans B de Francisco A Fogstad H Gupta N Laski L Lawn J Maliqi B Mason E Pitt C Requejo J Starrs A Victora CG Wardlaw T Countdown to 2015 decade report (2000-10): taking stock of maternal, newborn, and child survival Lancet 2010 375 2032 44 10.1016/S0140-6736(10)60678-2 20569843

[B2] Ravishankar N Gubbins P Cooley RJ Leach-Kemon K Michaud CM Jamison DT Murray CJL Financing of global health: tracking development assistance for health from 1990 to 2007 Lancet 2009 373 2137 69 19541038 10.1016/S0140-6736(09)60881-3

[B3] MEASURE DHS Demographic and Health Surveys http://www.measuredhs.com/(accessed December 2010)

[B4] UNICEF Statistics and monitoring. MICS http://www.unicef.org/statistics/index_24302.html (accessed December 2010)

[B5] MEASURE DHS Data Home http://www.measuredhs.com/accesssurveys/(accessed December 2010)

[B6] UNICEF Childinfo. Monitoring the situation of children and women http://www.childinfo.org/mics2_nationalreports.html (accessed December 2010)

[B7] Victora CG Fenn B Bryce J Kirkwood BR Co-coverage of preventive interventions and implications for child-survival strategies: evidence from national surveys Lancet 2005 366 1460 6 10.1016/S0140-6736(05)67599-X 16243091

[B8] Countdown Coverage Writing Group, on behalf of the Countdown to 2015 Core Group Countdown to 2015 for maternal, newborn, and child survival: the 2008 report on tracking coverage of interventions Lancet 2008 371 1247 58 18406859 10.1016/S0140-6736(08)60559-0

[B9] MEASURE DHS Download datasets http://www.measuredhs.com/LOGIN.cfm (accessed December 2010)

[B10] Granda P Blasczyk E XIII. Data Harmonisation. Cross-Cultural Survey Guidelines http://ccsg.isr.umich.edu/harmonization.cfm

[B11] ICSM International Committee on Surveying and Mapping. Data Framework Technical Sub-Committee and the ICSM Harmonised Data Model http://www.icsm.gov.au/icsm/harmonised_data_model/index.html

[B12] EUR-Lex Commission Regulation (EC) No 1205/2008 of 3 December 2008 implementing Directive 2007/2/EC of the European Parliament and of the Council as regards metadata (Text with EEA relevance) http://eur-lex.europa.eu/LexUriServ/LexUriServ.do?uri=CELEX:32008R1205:EN:NOT

[B13] Strötgen R Uher R ISSP DataWizard - Computer Assisted Merging and Archiving of Distributed International Comparative Data IASSIST 2000

